# Association of brain morphology and phenotypic profile in patients with unruptured intracranial aneurysm

**DOI:** 10.3389/fnagi.2023.1202699

**Published:** 2023-06-26

**Authors:** Jianyu Li, Zeming Tan, Xiaoping Yi, Yan Fu, Liping Zhu, Feiyue Zeng, Zaide Han, Zhanbing Ren, Yuanchao Zhang, Bihong T. Chen

**Affiliations:** ^1^Yangtze Delta Region Institute, University of Electronic Science and Technology of China, Huzhou, China; ^2^School of Life Sciences and Technology, University of Electronic Science and Technology of China, Chengdu, China; ^3^Department of Neurosurgery, Xiangya Hospital Central South University, Changsha, China; ^4^Department of Radiology, Xiangya Hospital Central South University, Changsha, China; ^5^Hunan Key Laboratory of Skin Cancer and Psoriasis, Xiangya Hospital Central South University, Changsha, China; ^6^Hunan Engineering Research Center of Skin Health and Disease, Xiangya Hospital Central South University, Changsha, China; ^7^National Clinical Research Center for Geriatric Disorders, Xiangya Hospital Central South University, Changsha, China; ^8^Department of Physical Education, Shenzhen University, Shenzhen, China; ^9^Department of Diagnostic Radiology, City of Hope National Medical Center, Duarte, CA, United States

**Keywords:** thalamic atrophy, local gyrification index, cortical gyrification, unruptured intracranial aneurysm, MRI

## Abstract

**Introduction:**

Studies have found a varying degree of cognitive, psychosocial, and functional impairments in patients with unruptured intracranial aneurysms (UIAs), whereas the neural correlates underlying these impairments remain unknown.

**Methods:**

To examine the brain morphological alterations and white matter lesions in patients with UIA, we performed a range of structural analyses to examine the brain morphological alterations in patients with UIA compared with healthy controls (HCs). Twenty-one patients with UIA and 23 HCs were prospectively enrolled into this study. Study assessment consisted of a brain magnetic resonance imaging (MRI) scan with high-resolution T1-weighted and T2-weighted imaging data, a Montreal Cognitive Assessment (MoCA), and laboratory tests including blood inflammatory markers and serum lipids. Brain MRI data were processed for cortical thickness, local gyrification index (LGI), volume and shape of subcortical nuclei, and white matter lesions.

**Results:**

Compared to the HCs, patients with UIA showed no significant differences in cortical thickness but decreased LGI values in the right posterior cingulate cortex, retrosplenial cortex, cuneus, and lingual gyrus. In addition, decreased LGI values correlated with decreased MoCA score (*r* = 0.498, *p* = 0.021) and increased white matter lesion scores (*r* = −0.497, *p* = 0.022). The LGI values were correlated with laboratory values such as inflammatory markers and serum lipids. Patients with UIA also showed significant regional atrophy in bilateral thalami as compared to the HCs. Moreover, the LGI values were significantly correlated with thalamic volume in the HCs (*r* = 0.4728, *p* = 0.0227) but not in the patients with UIA (*r* = 0.11, *p* = 0.6350).

**Discussion:**

The decreased cortical gyrification, increased white matter lesions, and regional thalamic atrophy in patients with UIA might be potential neural correlates of cognitive changes in UIA.

## Introduction

Unruptured intracranial aneurysm (UIA) is a serious cerebrovascular disease; up to 50% of patients who experience UIA rupture may die or suffer long-term disabilities (Towgood et al., 2004). In addition, patients with UIA may have cognitive, psychosocial, and functional impairments (Towgood et al., 2005; Bonares et al., 2016), resulting in diminished quality of life (van der Schaaf et al., 2002; Bonares et al., 2014). Existing studies of UIA have mainly focused on rupture risk assessment and treatment strategy. However, the potential neural mechanism underlying the cognitive changes in patients with UIA remains unknown. Notably, this knowledge can assist in decision-making for clinical management of UIA, such as the most appropriate operation time, follow-up strategy, and even necessary interventions to prevent cognitive impairment, so as to improve the quality of life of patients with UIA.

Neuroimaging studies have shown significant associations among brain morphological alterations such as the cortical gyrification pattern, white matter hyperintense lesions, and cognitive impairments in patients with neurodegenerative disease (Kievit et al., 2014; Prins and Scheltens, 2015; Tang et al., 2021). However, there is little information on whether patients with UIA may have distinct brain morphological patterns and whether these structural characteristics are associated with cognitive functioning. Notably, the risk factors for cerebrovascular disease, including hypertension, atherosclerosis, smoking, and neuroinflammation (Hokari et al., 2014; Kang et al., 2015), are known to affect brain structure (Wiseman et al., 2004; Etminan and Rinkel, 2016; Chesebro et al., 2020; Durazzo et al., 2020; Zeki Al Hazzouri et al., 2021). For instance, atherosclerosis and hypertension are associated with cortical thinning, brain atrophy, and white matter lesions (Wiseman et al., 2004; Durazzo et al., 2020). UIA and cerebrovascular disease share common risk factors, which may increase the occurrence and progression, and even rupture of UIAs. We therefore hypothesized that UIA may be associated with alterations in brain morphology and white matter integrity.

In the present study, we assessed brain morphological alterations in patients with UIA compared to healthy controls (HCs). Specifically, surface-based cortical thickness and local gyrification index (LGI) from brain magnetic resonance imaging (MRI) were analyzed to characterize morphological alterations of the cerebral cortex. In addition, the differences in volume and shape of the subcortical structures and white matter lesions were also examined for both the patients with UIA and HCs. We aimed to evaluate candidate brain structural alterations that could be structural correlates of cognitive functioning in patients with UIA.

## Materials and methods

### Subjects

Consecutive patients with UIA that was radiologically confirmed using brain computed tomography angiography (CTA) and digital subtraction angiography (DSA) scans were prospectively enrolled from January 1, 2019, to July 30, 2019. For comparison, age- and sex-matched HCs were recruited from the local community. Patients and HCs with a history of brain injury, epilepsy, neurodegenerative disease (such as Alzheimer’s disease and Parkinson’s disease), diabetes, and hypertension (moderate or severe) were excluded from this study. Radiological data, including routine brain MRI scans for both patients with UIA and the HCs, as well as CTA images and DSA images for the patients, were obtained with our standard scanning protocols. The main reasons for patients with UIA to receive a brain MRI scan included dizziness (*n* = 16, 76.2%), a history of transient ischemic attack (*n* = 6, 28.6%), and complaints of subjective memory loss (*n* = 10, 47.6%).

### Clinical and neuropsychological assessment

The demographic and clinical characteristics, radiological scans, and laboratory blood test results for patients with UIA were obtained from their medical records. The blood test results for the patients included the following: lymphocyte count, neutrophil-to-lymphocyte ratio (NLR), platelet count, serum lipids [total cholesterol (TC), total glyceride (TG), low-density lipoprotein (LDL), and high-density lipoprotein (HDL)], and inflammatory markers [tumor necrosis factor-alpha (TNF-α), interleukin 1 beta (IL-1β), interleukin 6 (IL-6), and interleukin 10 (IL-10)]. The patients with UIA were also assessed using the ELAPSS (Earlier subarachnoid hemorrhage, aneurysm Location, Age, Population, aneurysm Size and Shape) score for predicting the risk of UIA growth (Backes et al., 2017) and the PHASES (Population, Hypertension, Age, Size of aneurysm, Earlier subarachnoid aneurysm from another aneurysm, Site of aneurysm) score for management of UIA (Bijlenga et al., 2017).

All participants completed a set of questionnaires administered by a neuropsychologist within 72 hours of the planned hospital admission for treatment of UIA. These questionnaires for assessing cognition, depression, and anxiety level included the Montreal Cognitive Assessment (MoCA) (Nasreddine et al., 2005), the Patient Health Questionnaire-9 (PHQ-9)(Syed, 2013), and the General Anxiety Disorder-7 (GAD-7) (Spitzer et al., 2006). The HCs underwent the same questionnaire assessments as the patients with UIA. This study was approved by the Ethics Committee and Institutional Review Board in our institution (IRB No. 201812104). Written informed consent was obtained from all participants.

### Data acquisition

A T1-weighted MPRAGE (Magnetization Prepared – RApid Gradient Echo) sequence with whole-brain coverage was performed for all participants [acquisition matrix 240 mm × 256 mm × 176 mm, voxel size 1.0 mm × 1.0 mm × 1.0 mm, repetition time (TR)/echo time (TE) 8,020/50 ms, 30 interleaved slices with no gap] using the same Siemens MAGNETOM Prisma 3T MRI scanner. A Siemens parallel imaging implementation with iPAT = 2 was used to accelerate the data acquisition by a factor of 2, which reduced the acquisition time from 8 to 4 min. Additional imaging data, including the T2-weighted images and fluid attenuation inversion recovery (FLAIR) sequence, were also acquired for assessing white matter lesions.

### Cortical thickness and gyrification

The T1-weighted brain MRI data of each participant was processed using FreeSurfer^1^ to obtain the cortical thickness and LGI measurements. Specifically, the individual T1-weighted images were segmented to estimate the voxel-based gray/white matter boundary, which was triangulated to obtain a triangle-based gray/white matter boundary surface. This triangle-based gray/white matter surface was then topologically corrected to generate a refined gray/white matter surface (i.e., the white surface). The white surface was deformed outward with a deformable surface algorithm to generate the pial surface. The cortical thickness map of each participant was calculated by measuring the distance between the white surface and the pial surface using the T-average algorithm. The LGI map was obtained by measuring the ratio of the area of a circular region on the pial surface to the area of a corresponding circular region on the triangulated convex hull of the pial surface (Schaer et al., 2008). Prior to statistical analysis, the individual cortical thickness and LGI maps were resampled and further smoothed using a Gaussian kernel with a full-width-at-half-maximum of 20 and 10 mm, respectively.

### Subcortical volume and shape

Both volumetric and shape analyses were performed on the subcortical structures, including bilateral thalamus, hippocampus, amygdala, putamen, pallidum, and caudate. The volumes of these subcortical nuclei were extracted using the automated procedure for volumetric measures of the brain structures implemented in FreeSurfer. Shape analyses of these subcortical structures were conducted using the FIRST procedure in the FMRIB software library (FSL). Briefly, for each subcortical structure, FIRST created a surface mesh consisting of a set of vertices and triangles using a deformable mesh model. As the number of vertices for each structure was fixed, the spatial location of the corresponding vertices could be compared across participants. Given that the surface mesh of each participant resided in the native space and therefore may have different orientations and/or positions, we aligned the surface mesh of each participant to the mean surface in standard space to remove possible pose differences (rotation and translation) of each structure.

### White matter lesions

White matter lesions were assessed on the T2-weighted and FLAIR images according to a 4-point rating scale method proposed by Wahlund et al. (2001). Briefly, white matter changes on brain MRI were defined as hyperintense lesions ≥5 mm on T2-weighted, or FLAIR images. All ratings were performed by two experienced neuroradiologists independently (LZ and FZ, with 5 and 12 years of experience, respectively). If there was any discrepancy, a third senior neuroradiologist (ZH, with more than 25 years of experience) would be involved in the assessment and consensus would be reached through discussion among the three neuroradiologists.

### Statistical analyses

Vertex-wise contrasts of the cortical thickness and LGI maps between patients with UIA and HCs were performed using the SurfStat package^2^ in MATLAB. Specifically, for each vertex on the pial surface, we fitted a generalized linear model (GLM) with diagnosis, age, and sex as covariates. A vertex-wise *p* < 0.005 was used to define potential clusters of difference. Using random field theory (RFT), the resulting clusters were then corrected at the cluster level for multiple comparisons. The significance level for clusters was set at RFT-corrected *p* < 0.05.

Vertex-wise comparison of the spatial location in each subcortical structure between the two groups was performed using GLM in FSL with diagnosis, age, and sex as covariates. A non-parametric permutation test with 5,000 repetitions was then applied to obtain the group-wise statistics. The results were corrected for multiple comparisons using threshold-free cluster enhancement (TFCE). The significance level was set at TFCE-corrected *p* < 0.05.

Pearson’s correlation coefficient was used to determine the association between morphological alteration and clinical data. Spearman’s rank correlation coefficient was used to determine the association between morphological alteration and the white matter (WM) lesions.

## Results

### Demographic, clinical, and neuropsychological characteristics

A total of 21 patients with UIA and 23 HCs were included in this study. The demographic information, clinical data, and laboratory values are presented in Table 1. There were no significant differences in age, sex, and body mass index between the patients with UIA and the HCs. However, the patients with UIA had lower MoCA scores than HCs (*p* < 0.01). There were no significant differences between the two groups in the PHQ-9 score for depression (*p* = 0.513) and GAD-7 score for anxiety (*p* = 0.759).

**TABLE 1:** Participant information.

	Patients with UIA (*n* = 21)	Healthy controls (*n* = 23)	*p*-Value
**Demographic characteristics**			
Age (years)	55.0 (48.5, 61.0)	55.0 (48.0, 57.0)	0.54
Sex (male/female)	5/16	5/18	0.87
Body mass index	22.76 (21.33, 26.27)	23.14 (21.64,25.20)	0.63
Education (years)	9.0(6.00, 12.75)	9.0(6.75, 12.00)	0.82
**Neuropsychological questionnaires**			
Cognitive function, MoCA	24 (20, 27)	27 (26, 28)	<0.01
Depression, PHQ-9	45 (35, 49)	43 (35, 47)	0.513
Anxiety, GAD-7	37 (32, 44)	37 (32, 42)	0.759
**UIA data**			
ELAPSS score	10 (5, 17)	–	
PHASES score	3 (0, 6)	–	
Location of aneurysm			
ACA	9 (42.86%)	–	
PCA (left)	4 (19.05%)	–	
PCA (right)	2 (9.52%)	–	
MCA (left)	4 (19.05%)	–	
MCA (right)	2 (9.52%)	–	
**Size of aneurysm (mm)**			
≤3.9	3 (14.29%)	–	
4.0∼6.9	12 (57.14%)	–	
7.0∼12.9	3 (14.29%)	–	
13∼24.9	2 (9.52%)	–	
≥25.0	1 (4.76%)	–	
**Laboratory data**			
Routine blood markers		–	
Lymphocyte count	1.80 (1.30, 2.15)	–	
Neutrophil count	3.30 (2.85, 4.60)	–	
NLR	2.18 (1.34, 2.75)	–	
Platelet	202.0 (180.0, 230.5)	–	
Inflammatory markers		–	
TNF-α	3.83 (2.86, 6.76)	–	
IL-1β	1.74 (1.20–3.85)	–	
Serum lipids		–	
TC	4.89 (3.87, 5.95)	–	
HDL	1.05 (0.81, 1.42)	–	

Continuous variables are presented as median (interquartile range). Between-group differences in these variables were tested using the Mann–Whitney U test or Chi-square test. ACA, anterior communicating artery; PCA, posterior communicating artery; MCA, middle cerebral artery; NLR, neutrophil-to-lymphocyte ratio; TNF-α, tumor necrosis factor-alpha; IL-1β, interleukin 1 beta; TC, total cholesterol; HDL, high-density lipoprotein.

### Cortical thickness and LGI

**FIGURE 1:**
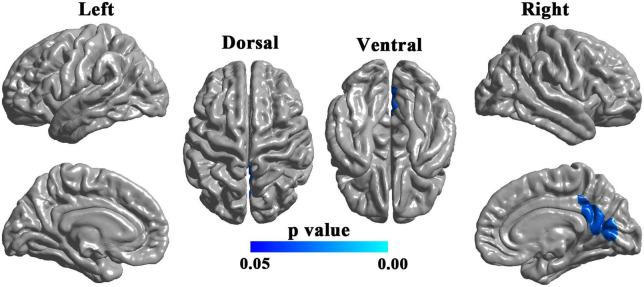
Brain regions with decreased local gyrification index (LGI) in patients with unruptured intracranial aneurysm. The blue-colored brain region in the right lower panel indicates a cluster with significantly reduced LGI in the right hemisphere, involving the right posterior cingulate cortex, retrosplenial cortex, cuneus, and lingual gyrus. The results were random field theory (RFT)-corrected. The color bar denotes the RFT-corrected *p*-value.

There was no significant difference in cortical thickness between the two groups (RFT-corrected *p* > 0.05). Compared with the HCs, the patients with UIA showed significantly reduced LGI in a cluster in the right hemisphere, involving the right posterior cingulate cortex, retrosplenial cortex, as well as cuneus, and lingual gyrus (cluster size = 5,179 vertices, peak *t* value = 3.4616, peak Talairach coordinates: *x* = 6.42, *y* = −60.37, *z* = 11.92) (Figure 1).

### Associations between LGI and clinical data

**FIGURE 2:**
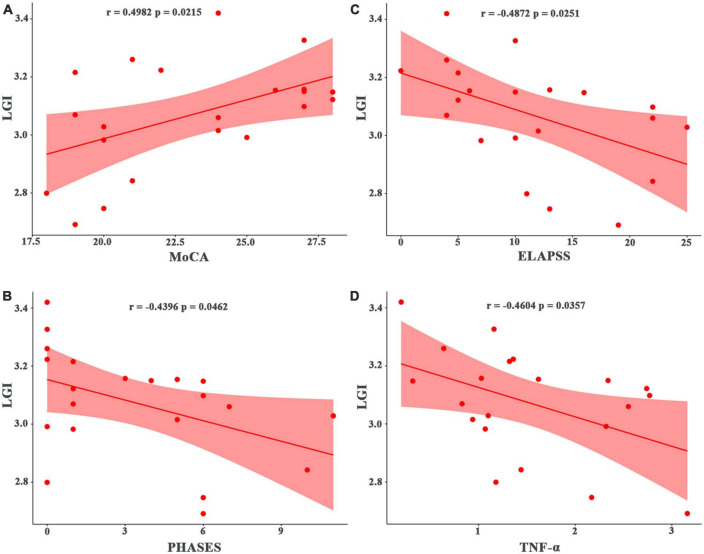
Correlation of local gyrification index (LGI) with cognitive, clinical, and laboratory test scores in patients with unruptured intracranial aneurysm. **(A)** Correlation with the MoCA score. **(B)** Correlation with the PHASES aneurysm score. **(C)** Correlation with the ELAPSS aneurysm score. **(D)** Correlation with inflammatory marker (TNF-α). MoCA, Montreal Cognitive Assessment; PHASES, Population, Hypertension, Age, Size of aneurysm, Earlier subarachnoid aneurysm from another aneurysm, Site of aneurysm; ELAPSS, Earlier subarachnoid hemorrhage, aneurysm Location, Age, Population, aneurysm Size and Shape; TNF-α, tumor necrosis factor-alpha.

The LGI values for the significant cluster were positively correlated with the MoCA scores (*r* = 0.498, *p* = 0.021) (Figure 2A) but not significantly correlated with the depression PHQ-9 scores or anxiety GAD-7 scores (*p* > 0.05). The LGI values for the significant cluster were negatively correlated with the PHASES and ELAPSS aneurysm scores in patients with UIA (*r* = −0.440, *p* = 0.046; *r* = −0.487, *p* = 0.025; respectively) (Figures 2B, C).

There were significant correlations between the LGI values and laboratory data. In patients with UIA, the LGI values were negatively correlated with lipid values such as TC (*r* = −0.048, *p* = 0.028) but positively correlated with HDL levels (*r* = 0.539, *p* = 0.012). Also, in patients with UIA, the LGI values were negatively correlated with blood PLT counts (*r* = 0.546, *p* = 0.010) and inflammatory markers such as TNF-α (*r* = −0.460, *p* = 0.036) (Figure 2D) and IL-1β (*r* = −0.462, *p* = 0.035). The LGI values were also negatively correlated with the white matter lesions (*r* = −0.497, *p* = 0.022) in patients with UIA.

### Subcortical volume and shape

**FIGURE 3:**
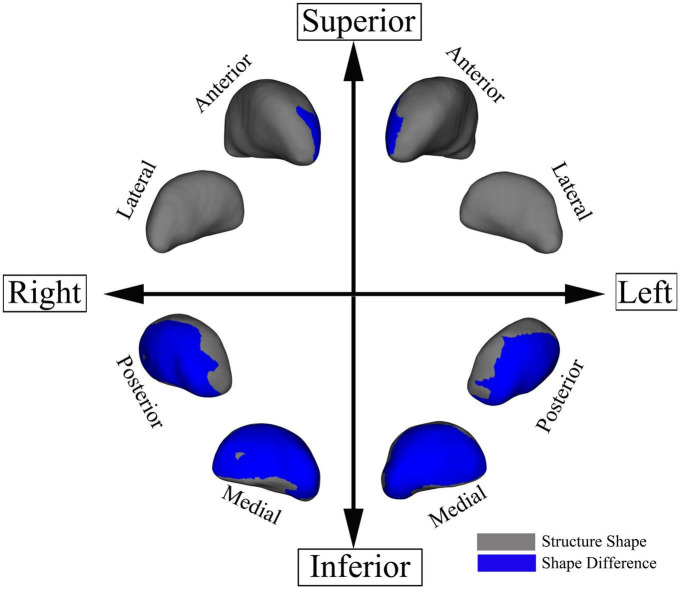
Regional thalamic atrophy in patients with unruptured intracranial aneurysm. The blue-colored areas in the thalamus indicate the regional atrophy in the medial and posterior aspects of the thalamus. The results were threshold-free cluster enhancement-corrected for multiple comparisons.

Patients with UIA showed significant bilateral regional atrophy in the medial and posterior parts of the thalami as compared to the HCs (TFCE-corrected *p* < 0.05) (Figure 3). However, there was no significant difference in the total thalamic volume between the two groups (*p* = 0.257). There were no significant differences in the shape and volume of other subcortical structures between the two groups.

**FIGURE 4:**
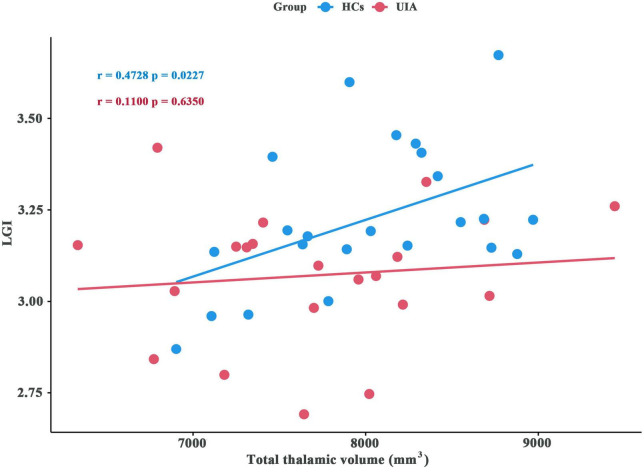
Association between local gyrification index (LGI) and total thalamic volume for patients with unruptured intracranial aneurysm (UIA) and healthy controls (HCs). The blue circles indicate the data for the HCs and the red circles indicate the data for patients with UIA.

The LGI values for the significant cluster were positively correlated with the total thalamic volumes (*r* = 0.4728, *p* = 0.0227) in the HCs but not in patients with UIA (*r* = 0.11, *p* = 0.6350) (Figure 4).

### White matter lesions

For the patients with UIA, 5 out of 21 patients were rated as 1 for white matter lesion (i.e., with 1 focal lesion >5 mm) (23.8%), 1 out of 21 patients was rated as 2 (i.e., with >1 focal lesion) (4.7%), and 15 out of 21 patients were rated as 0 (i.e., with no lesion) (71.4%). For the HCs, only 1 patient was rated as 2, while the remaining HCs were all rated as 0 (95.7%). The mean white matter lesion score in patients with UIA was significantly higher than that of the HCs (*p* = 0.038).

## Discussion

In this study, we found a distinct pattern of decreased gyrification in a specific right posterior cortical region, which was correlated with cognitive testing scores, aneurysm assessment scores, blood lipid values, and inflammatory markers in patients with UIA. In addition, we found shape alterations in the thalamus and increased white matter lesions in patients with UIA. These results support the notion of neural correlates for cognitive functioning in patients with UIA.

Our study identified decreased gyrification in the key hubs of the default mode network (DMN) including the right posterior cingulate cortex and retrosplenial cortex in patients with UIA (Fransson and Marrelec, 2008; Vann et al., 2009). The DMN has been implicated in cognitive function, social cognition, memory, and emotional processing (Raichle et al., 2001; Raichle, 2015; Fox et al., 2017; Satpute and Lindquist, 2019). Indeed, patients with UIA have impaired cognitive function, visual attention, psychosocial functioning, delayed recall, and psychiatric symptoms such as depression and anxiety (van der Schaaf et al., 2002; Towgood et al., 2005; Bonares et al., 2014, 2016). It is therefore reasonable to speculate that our observed LGI decrease may be related to the cognitive dysfunction in patients with UIA. This speculation was supported by our finding of a positive correlation between the LGI values and MoCA scores in patients with UIA. In addition, the lack of a significant correlation between LGI and the scores for depression and anxiety in our study was also understandable, as these symptoms were usually transient and difficult to assess (Yamashiro et al., 2007; Su et al., 2014). Furthermore, our study showed decreased LGI in the visual cortices (lingual gyrus and cuneus) in patients with UIA, which may account for the visual impairments and the associated cognitive dysfunction in these patients (Bonares et al., 2016).

Prior studies have reported that the risk factors for UIA, such as smoking and hypertension, may affect thalamic volume (Jennings et al., 2012; Liao et al., 2012; Hanlon et al., 2016), indicating that the thalamus in patients with UIA is vulnerable to alterations. However, we found no significant differences in total thalamic volume between the patients and HCs. This finding was not a surprise, as brain structures such as the thalamus may compensate, with some subregions decreasing in volume and some increasing in volume to maintain a constant total volume for normal function. In our shape analysis of subcortical nuclei, we found the expected significant bilateral regional atrophy in the medial and posterior parts of the thalami. The underlying mechanism for the thalamic shape modification was not clear. We speculate it may be related to the overall function of the thalamus as a relay station for transferring information to the cortex (Wolff and Vann, 2019). The risk factors for UIA, such as hypertension, neuroinflammation, and arteriosclerosis, may predispose the brain to injury and the thalamus may be particularly sensitive to the insult and undergo internal shape modification. For instance, the mediodorsal thalamus is involved in cognition, especially learning and memory (Wolff and Vann, 2019), and therefore it was not surprising that our study showed a reduction in the mediodorsal thalamus and diminished cognitive function in patients with UIA. Our study also showed a lack of correlation between gyrification and the total thalamic volume in patients with UIA but the correlation was present in the HCs. Considering the extensive reciprocity of projections between cortical and thalamic areas in the thalamocortical circuits (Behrens et al., 2003; Dong et al., 2012; Wei et al., 2019), this finding implies that thalamocortical pathways may be disrupted in patients with UIA.

Our study showed a distinct gyrification pattern, regional thalamic atrophy, and white matter lesions in patients with UIA, implying that patients with UIA undergo neuroplasticity to maintain function. First, the risk factors for UIA can have detrimental effects on white matter integrity and contribute to the occurrence of white matter lesions (Wersching et al., 2010; Ederle et al., 2013; Prins and Scheltens, 2015; Chesebro et al., 2020). The affected white matter may alter the tension along the underlying white matter tracts, which connect the subcortical structures to the cerebral cortex, resulting in cortical changes (Van Essen, 1997). Specifically, we speculate that the tension of the thalamocortical tract in patients with UIA may be altered by white matter lesions, leading to diminished connectivity to the cortex and contributing to the decreased gyrification. The cortex may also reciprocally affect the thalamus through inter-connectivity, resulting in regional thalamic atrophy. This speculation was supported by our observation of a correlation between the LGI values and the white matter lesions. Secondly, we identified a correlation between the cortical LGI values and the risk factor profiles, including inflammatory markers and serum lipids, which are collected as part of atherosclerosis evaluation in patients with UIA. We also identified a correlation between the LGI values and aneurysm assessment scores (ELAPSS and PHASES) in patients with UIA. These results indicate that the risk factor profile was associated with alterations in the cortical morphology in addition to white matter integrity. The presence of changes in both cortical morphology and white matter in patients with UIA implies potential compensatory neuroplasticity. However, the exact neural mechanism underlying the structural alterations remains unclear and further research is needed to understand the implications of such changes on cognitive function in patients UIA.

There were a few limitations to this study. First, this study had a relatively small sample size, which limited our ability to perform subgroup analyses based on the location and size of the aneurysms. Future studies with larger sample size are warranted to explore the potential impact of these factors on brain morphology and clinical manifestation in patients with UIA. Second, the patients in our study were recruited prior to treatment, but all patients subsequently underwent treatment for their UIAs either through craniotomy for aneurysm clipping or endovascular coiling. Therefore, the patients in our study may represent a subset of UIA patients with more serious UIAs, a higher risk of UIA rupture, and greater cognitive dysfunction than patients who did not undergo treatment. Third, we could not quantitatively assess the detailed white matter microstructural abnormalities across the whole brain or within the thalamocortical network due to the lack of diffusion tensor imaging data. Lastly, we could not characterize the longitudinal brain structural changes in these patients due to the cross-sectional design of this study and lack of follow-up data on their cognitive function after the UIA treatment. Despite the limitations, this study has its merit as it generated promising preliminary data on brain morphological alterations and their associations with cognitive function. The results are expected to motivate further research on the effects of UIA on brain and cognition.

## Conclusion

In summary, we found decreased cortical gyrification, increased white matter lesions, and regional thalamic atrophy in patients with UIA. Our results imply that these brain structural alterations might be the neural correlates underlying the functional alterations in patients with UIA.

## Data availability statement

The raw data supporting the conclusions of this article will be made available by the authors, without undue reservation.

## Ethics statement

The studies involving human participants were reviewed and approved by the Ethics Committee and Institutional Review Board in Xiangya Hospital. The patients/participants provided their written informed consent to participate in this study.

## Author contributions

JL, YZ, and XY performed the material preparation, data collection, and analysis. JL wrote the first draft of the manuscript. All authors contributed to the study conception and design, commented on previous versions of the manuscript, and read and approved the final manuscript.

## Footnotes

1
https://surfer.nmr.mgh.harvard.edu/

2
http://www.math.mcgill.ca/keith/surfstat/
